# Development of a Bioelectrochemical System as a Tool to Enrich H_2_-Producing Syntrophic Bacteria

**DOI:** 10.3389/fmicb.2019.00110

**Published:** 2019-02-05

**Authors:** Juan J. L. Guzman, Diana Z. Sousa, Largus T. Angenent

**Affiliations:** ^1^Biological and Environmental Engineering Department, Cornell University, Ithaca, NY, United States; ^2^Laboratory of Microbiology, Wageningen University and Research, Wageningen, Netherlands; ^3^Centrum for Applied Geosciences, University of Tübingen, Tübingen, Germany

**Keywords:** bioelectrochemistry, syntrophy, syntrophic bacterium, methanogenic partner, hydrogen, *Syntrophus*

## Abstract

Syntrophic microbial partnerships are found in many environments and play critical roles in wastewater treatment, global nutrient cycles, and gut systems. An important type of syntrophy for the anaerobic conversion of carboxylic acids is H_2_ syntrophy. In this type of microbial partnership, dissolved H_2_ is produced by a bacterium and rapidly consumed by an archeon (methanogen), resulting in methane gas. This is referred to as interspecies H_2_ transfer, and some conversions rely on this mechanism to become thermodynamically feasible. For this reason, syntrophic partners are often not possible to separate in the lab, which hampers the full understanding of their physiology. Bioelectrochemical systems (BESs) may show promise to ultimately separate and study the behavior of the syntrophic bacterium by employing an abiotic H_2_ oxidation reaction at the anode, actively removing dissolved H_2_. Here, we performed a proof-of-concept study to ascertain whether an H_2_-removing anode can: (1) provide a growth advantage for the syntrophic bacterium; and (2) compete with the methanogenic partner. A mathematical model was developed to design a BES to perform competition experiments. Indeed, the operated BES demonstrated the ability to provide a growth advantage to the syntrophic bacterium *Syntrophus aciditrophicus* compared to its methanogenic partner *Methanospirillum hungatei* when grown in co-culture. Further, the BES provided the never-before isolated *Syntrophomonas zehnderi* with a growth advantage compared to *Methanobacterium formicicum*. Our results demonstrate a potential to use this BES to enrich H_2_-sensitive syntrophic bacteria, and gives prospects for the development of an effective method for the separation of obligate syntrophs.

## Introduction

Syntrophic microbial partnerships seem to exist wherever substrates are degraded under anaerobic conditions; they are particularly present in anaerobic digesters, soil, swamps, oil tanks, hot springs, and in the guts of various mammals (Dolfing et al., 2007; Morris et al., 2013). Syntrophic microbial partnerships play important roles in global carbon, nitrogen, and sulfur cycles, yet their exact role and level of impact in these environments has not been fully understood because of the difficulty in culturing these microbes in laboratory settings (Rappé and Giovannoni, 2003). A considerable portion of syntrophic microbial partnerships remain unisolated (Morris et al., 2013). Syntrophic microbial partnerships consist of two tightly linked microbes that depend on each other to improve the energetics of their metabolism. For one type of syntrophic microbial partnership, a H_2_-producing bacterium grows with a H_2_-consuming archaeon (methanogen). For the syntrophic bacterium alone, its anaerobic substrate oxidation would not be thermodynamically feasible due to ever-increasing H_2_ concentrations, leading to feedback inhibition of syntrophic growth. In a partnership with the methanogen, however, H_2_ is consumed to very low concentrations and converted into methane at high rates, providing both microbes with growth benefits (McInerney et al., 2008). This relationship is referred to as interspecies H_2_ transfer. It is generally accepted through studying syntrophic metabolism in partnerships and through modeling that syntrophic bacteria are extremely sensitive to the presence of H_2_ in their environments. With the extremely low concentration of H_2_ predicted to be necessary to provide the syntrophic bacterium with a growth advantage compared to the methanogenic partner (below 62 nM), few engineered alternatives have been developed to ultimately isolate syntrophic bacteria (Boone et al., 1989; Schink and Stams, 2006; McInerney et al., 2009).

Attempts have been made to develop approaches to systematically culture, enrich, or isolate these syntrophic bacteria, particularly with novel bioreactor designs and specialized media. While some approaches have demonstrated success in a select number of cases, an application past the proof-of-concept has not been reported or led to a widely known microbiological tool. In one instance, a chemostat was aggressively sparged with anaerobic gas to strip H_2_, which provided an improved growth environment and resulted in a higher H_2_ production rate for the syntrophic bacterium in a co-culture (Valentine et al., 2000). The apparatus also led to higher productivities of the syntrophic microbial partnership when the anaerobic gas was flowing, indicating that H_2_ build-up in normal environments reduced syntrophic activity. The same apparatus was also used to study the maximum H_2_ production of a syntrophic bacterium in a pure culture by maintaining extremely low H_2_ concentrations, which was estimated at 10 pM (approximately 0.25 ppb) (Adams et al., 2006). In another instance, palladium catalyst was added to the liquid and gas phase of a bioreactor to quickly react with short-chain carboxylic acids and H_2_, which were produced *via* fermentation, resulting in the production of long-chain carbons (Mountfort and Kaspar, 1986). This study was successful in limiting methanogenesis by diverting H_2_, but did not demonstrate its ability to enrich for a syntrophic bacterium.

Bioelectrochemical systems (BESs) have been developed for numerous environmental and biotechnological applications such as waste treatment, chemical and energy production, and biosensing and Biocomputing (Cheng et al., 2009; Fornero et al., 2010; TerAvest et al., 2011; Angenent and Rosenbaum, 2013; Gavazza et al., 2015). BESs are also ideal systems for studying microbes by altering the electrochemical activity of the environment. By controlling the electrode potential, microbes that are capable of direct or mediated electron transfer can be studied in controlled environments to observe their electric current production rates, metabolic activity, and growth. In essence, BESs are a valuable microbiological tool that can use a real-time electrical signal as a proxy for the microbial activity (Venkataraman et al., 2010).

Here, we evaluated the potential use of a BES as a tool to enrich syntrophic bacteria at the working electrode (anodic function) in a first proof-of-concept competition effort to ascertain whether such a BES has merit to perform further studies. BESs typically use three electrodes (i.e., working, counter, and reference), which allow the potential at the working electrode to be controlled to, for example, sustain an abiotic H_2_ oxidation reaction. This would create an ideal environment in the BES with a very low H_2_ concentration to successfully compete with the methanogenic partner. To drive this H_2_ oxidation reaction, a catalyst, such as platinum at the working electrode (anode), is required to promote the reaction, and the potential of the working electrode theoretically only needs to be maintained slightly above 0 V vs. the standard H_2_ electrode (SHE) (Cheng et al., 2005). Most studies evaluating H_2_ oxidation maintained a potential of +300 mV (vs. Ag/AgCl), which provided sufficient over-potential to overcome system losses. Here, we show a proof-of-concept of a BES for the enrichment of H_2_ syntrophic bacteria.

For the development of our BES, we organized the work into four steps: (1) we modeled a maximum threshold H_2_ concentration that should be maintained in a BES to deliver a growth advantage for the syntrophic bacterium compared to the methanogenic partner, (2) we modeled the ability for BESs with different distances away from the electrode ranging from 0.1 to 100 mm to oxidize H_2_ at this threshold H_2_ concentration within *in silico* competition experiments, (3) we built a BES based on the modeling results, and (4) we performed microbial competition experiments by inoculating two co-cultures containing syntrophic bacteria to prove the concept within one bioreactor study with its control. First, we inoculated a co-culture of *Syntrophus aciditrophicu*s and *Methanospirillum hungatei*. This co-culture was tested as a proof-of-concept: the syntrophic bacterium, *S. aciditrophicus*, has already been separated from the methanogen, *M. hungatei*, which can also grow in pure culture (Jackson et al., 1999; McInerney et al., 2007). By combining these two microbes back into a co-culture, and then demonstrating enrichment of the syntrophic bacterium compared to the methanogenic partner, we would have a strong indication that the BES was operating as we had intended. Second, we inoculated an obligately syntrophic co-culture of *Syntrophomonas zehnderi* and *Methanobacterium formicicum* (Sousa et al., 2007). We performed this experiment as an indication that the BES could enrich for a syntrophic bacterium in a co-culture that had never been separated through typical culturing efforts.

## Materials and Methods

### Growth Media and Cultures

*Syntrophus aciditrophicus* (DSM 26646) was cultured in a growth medium recipe including 8 mM crotonic acid as a carbon source in pure culture, and with a 80%:20% N_2_:CO_2_ headspace (Jackson et al., 1999; Mouttaki et al., 2008). *M. hungatei* (ATCC 27890) was cultured in ATCC Medium 2487 with dissolved 2.5 mM formic acid and 20% H_2_ at 1.01 × 10^5^ kPa (1 atm) in the headspace (Patel et al., 1976; Jackson et al., 1999). To prepare a 1:1 co-culture of *S. aciditrophicus* and *M. hungatei* as the inoculum, we first prepared a stock for each culture to a similar copy number by dilution with the specific growth medium, and then mixed the stocks together. We had used qPCR to correlate DNA to the copy number. *M. formicicum* was cultured in ATCC Medium 1045 with 20% H_2_ at 1.01 × 10^5^ kPa in the headspace (Sousa et al., 2007). *S. zehnderi* does not exist as a pure culture, but as a co-culture with *M. formicicum*; inoculations were “lawned” on *M. formicicum* with 0.5 mM oleic acid. Thus, the *S. zehnderi*-containing co-culture was inoculated into fresh *M. formicicum* medium with the headspace sparged with 80%:20% N_2_:CO_2_ to remove remaining H_2_ gas. All cultures studied here were grown in anaerobically prepared sterile serum bottles, and sterile practices were used during all sampling and inoculations. Growth rate terms (Supplementary Table S1) were obtained from the linear region of the growth curve obtained within serum bottle growth tests.

### Modeling

This study involved two mathematical modeling efforts. The first model determined the maximum H_2_ concentration (threshold) that can provide a growth advantage to the syntrophic bacterium compared to the methanogenic partner. We utilized a mixture of published data and experimental growth data with serum bottles to calculate the maximum growth rate of the syntrophic bacterium and the methanogenic partner based on Monod growth kinetics (Supplementary Material S1, Supplementary Equations S1, S2, and Supplementary Table S1). The second model determined the maximum distance away from the electrode that this threshold can be reached within a reasonable time frame. This model was generated as a 1-dimentional space in COMSOL Multiphysics^TM^, using a system of equations (Supplementary Equations S3–S6) and constants (Supplementary Table S1). This model accounted for H_2_ diffusion and oxidation, growth of the syntrophic bacterium and the methanogenic partner, and H_2_ production and consumption by the microbes and electrodes; which was evaluated through time and in batch mode. The H_2_ oxidation reaction was performed as a heterogeneous reaction at the working electrode surface, rather than in the liquid bulk, which has been reported in other BES modeling efforts. Therefore, concentration gradients of H_2_ and microbes could be evaluated.

### Bioelectrochemical System

**FIGURE 1:**
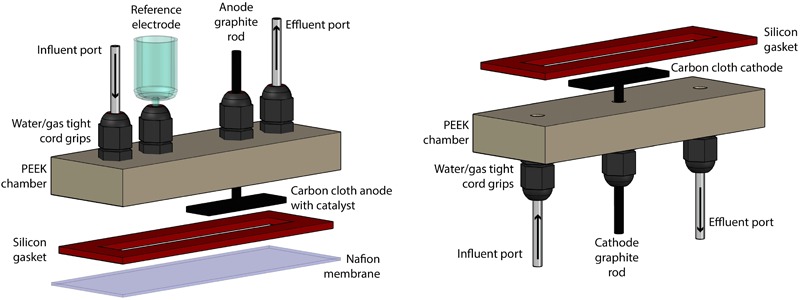
Rendering of the construction of the BES, which was separated into anode section **(left)** and cathode section **(right)**.

The bioelectrochemical system (BES) consisted of two identical chambers that were machined from polyether ether ketone (PEEK, McMaster-Carr, Elmhurst, IL, United States) (Figure 1). These chambers contained liquid- and gas-tight cord grips to hold the working, reference, and counter electrodes in a water-and gas-tight fashion. The chambers also contained inlet and outlet ports for sampling. Two aluminum-clamping plates were machined to hold the two chambers on either side of a Nafion membrane (Nafion 117, FuelCellsEtc, United States) and used silicon rubber gaskets (McMaster-Carr, Elmhurst, IL, United States) to make water-tight seals. The internal volume of the BES was contained within a cavity cut out of the silicon rubber gasket, which was 8.8 cm × 1.27 cm and 2.33 mm thick. The BES was assembled and autoclaved, prepared anaerobically and sterilely, and operated within a custom anaerobic enclosure (80%:20% N_2_:CO_2_) at 37°C (Supplementary Figure S1). For all BES experiments, two identical systems were operated at the same time for simultaneous test and control BESs. The difference between the test and control systems was that the test system had active electrodes connected to a potentiostat (VSP, Biologic Science Instruments, Claix, France) and operated chrono-amperometrically at + 0.3 V (vs. Ag/AgCl), while the control system electrodes remained unconnected.

### Electrodes

The counter electrode consisted of a 2 cm × 1 cm piece of carbon cloth with 1 mm thickness (PANEX^®^ 30-PW06, Zoltek Corp, St Louis, MO, United States) attached to a 4.76-mm diameter fine extruded graphite rod (Graphite Store, Buffalo Grove, IL, United States), using a drop of conductive carbon cement (CCC, Electron Microscopy Sciences, Hatfield, PA, United States), as published previously (Guzman et al., 2017). The working electrode was prepared identically, with the addition of 20 μL⋅cm^-2^ of a paste consisting of 1.24⋅10^-1^ g Pt/Carbon powder per 1 mL of Nafion solution (Sigma-Aldrich, St. Louis, MO, United States) (Cheng et al., 2005). The paste was applied by pipette and allowed to dry in an oven overnight at 80°C. Critically, *prior* to any experiments, the electrodes were vacuumed in DI water for at least 15 min to expel any O_2_ held within the fine surfaces of the carbon cloth.

### Microbial Analysis

Genomic DNA was extracted from the entire pellet from the sample using the PowerSoil kit (MO BIO Laboratories Inc., Carlsbad, CA, United States). For DNA extracted from electrodes, the carbon cloth piece was detached from the graphite rod and inserted into the PowerSoil kit, and the procedure remained the same. Modifications to the protocol, which was provided by the manufacturer, include using custom bead tubes with sterilely prepared 0.1-mm and 0.5-mm diameter silica/zirconia beads. Cell lysis was achieved by bead-beating at 3550 oscillations min^-1^ for 45 s. DNA quantification was performed in triplicate using SYBR Green FastStart Universal qPCR Mix (Roche Molecular Systems, Inc., Indianapolis, IN, United States) and Fast SYBR Green Master Mix (Applied Biosystems, Waltham, MA, United States). Primer sequences were obtained from literature (Supplementary Table S2). qPCR standard curves were determined for each strain (Supplementary Figures S2–S5). The standards were obtained using primers designed for each strain using NCBI Primer BLAST (Supplementary Table S2) with the BLAST Sequence Analysis Tool (Madden, 2002).

### Growth Analysis

Optical density for cell growth (OD_600_) and pH were measured immediately after sampling. OD_600_ was measured in a spectrophotometer at 600 nm with a path length of 1 cm. Samples used for carboxylic acid analysis were centrifuged and stored frozen at -20°C until analysis by gas chromatography, as previously reported (Usack and Angenent, 2015).

## Results

### Maximum H_2_ Concentration to Select for Syntrophic Bacteria

**FIGURE 2:**
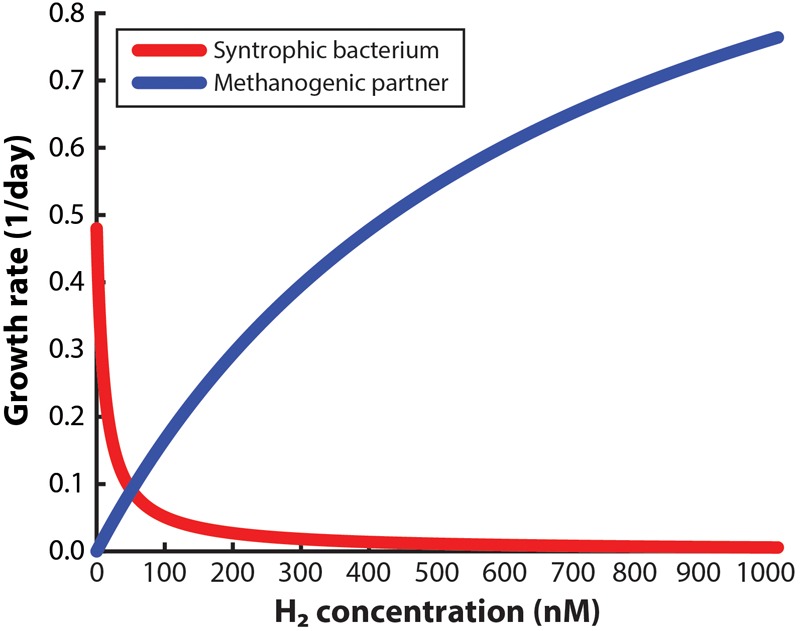
Monod-based growth rates for the syntrophic bacterium, *Syntrophus aciditrophicus*, and the methanogenic partner, *Methanospirillum hungatei*, which we evaluated at different H_2_ concentrations.

To design a system to enrich a syntrophic bacterium from a co-culture, the environment must be managed so that the syntrophic bacterium is given a growth advantage in the entire working volume compared to the methanogenic partner. For this approach to succeed, not only should the dissolved H_2_ concentration in the bulk liquid be low enough to allow the syntrophic bacterium to grow alone, but it should also be low enough to slow down the growth of the methanogenic partner. Many studies have claimed that very low H_2_ concentrations will be necessary (Boone et al., 1989; Hallenbeck and Benemann, 2002; Schink and Stams, 2006), yet a maximum allowable concentration (threshold) has not been reported to accomplish both goals. Here, we have determined the maximum H_2_ concentration for the syntrophic bacterium *S. aciditrophicus* to outgrow *M. hungatei* when in a co-culture. From this analysis, we determined that at a H_2_ threshold of 51 nM in the bulk liquid, the syntrophic bacterium and the methanogenic partner have identical growth rates (i.e., intersection point in Figure 2). Thus, at concentrations below 51 nM the growth of the syntrophic bacterium would dominate compared to the methanogenic partner and *vice versa*. We had to assume that this threshold H_2_ concentration is similar for both co-cultures that we used in this study, because growth data for the never-isolated syntrophic bacterium is not available. We further used this 51 nM threshold as an engineering target for further modeling and engineering design.

### Maximum Distance Away From the H_2_-Oxidizing Electrode in a BES

We then developed a model to evaluate the maximum distance away from the electrode to provide such a growth advantage to a syntrophic bacterium growing in a co-culture within a BES operating in batch mode. We predicted the H_2_ concentration gradient at distances of 0.1, 1, 10, and 100 mm away from the electrode in a BES that is operated in batch mode. The assumed heterogeneous reaction at the electrode would oxidize H_2_ quickly at the region closest to the electrode, which would establish a concentration gradient in the system, driving H_2_ diffusion toward the electrode from areas in the bulk liquid farther away where the syntrophic bacterium would produce H_2_.

Four different distances from the electrode surface were evaluated: 0.1, 1, 10, and 100 mm. The 0.1-mm distance was chosen to simulate the environment that would be observed in a microfluidic system. For the 1-mm distance, the H_2_ profile was evaluated at the 0.1 and 1 mm edge of the system. For the 10-mm distance, the H_2_ profile was evaluated at the 0.1, 1, and 10-mm edge of the system. Finally, for the 100-mm distance system, the H_2_ profile was evaluated at the 0.1, 1, 10, and 100 mm edge. We then ran the model and determined the exposure time necessary to reach the 51 nM H_2_ threshold from an initial value of 1000 nM. We assumed an exposure time below 15 min to be acceptable for the syntrophic bacteria to remain above 51 nM; exposure times greater than 15 min would be unsuitable for culturing syntrophic bacteria.

**FIGURE 3:**
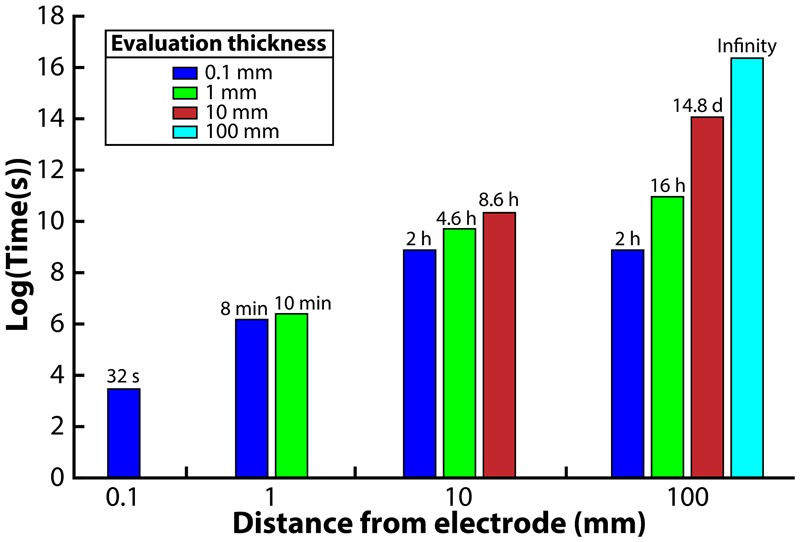
Required time to oxidize H_2_ to meet the 51 nM threshold for each reactor distance, evaluated at different distances from the anode electrode.

When we evaluated the shortest distance (0.1-mm), the electrode was able to oxidize the H_2_ in the entire volume within 32 s to reach the 51 nM H_2_ threshold (Figure 3). The 1-mm distance, on the other hand, was able to oxidize the H_2_ down to the target threshold for 0.1 and 1 mm within 8 and 10 min, respectively, which is acceptable with our assumed 15-min limit. The large difference in exposure times at a 0.1-mm distance from the electrode for these two distances shows that the H_2_ profile in the entire system is highly affected by the bulk liquid that is furthest away from the electrode. For the 10-mm distance, we determined that the 0.1, 1, and 10 mm distances required 2, 4.6, and 8.6 h to reach the 51 nM threshold, making this system too thick for culturing syntrophic bacteria based on our assumed maximum exposure time limit (Figure 3). When we finally evaluated the model for the 100-mm distance, achieving a H_2_ concentration low enough to favor syntrophic bacterial growth was not possible in the entire system (Figure 3). At the edge of the system, which was 100 mm away from the electrode, the H_2_ concentration never reached the 51 nM threshold even after a month of operation. The H_2_ concentration evaluated at a 0.1, 1, and 10 mm distance required 2 h, 16 h, and 14.8 days to achieve the threshold, respectively.

These results may provide some insight to the reason why BESs have not been studied previously for enriching syntrophic bacteria. These predictions indicated that a much smaller system would be necessary to culture the syntrophic bacterium successfully compared to conventional BESs, particularly if trying to encourage enrichment in the bulk in addition to the electrode surface. Our modeling efforts showed that large volumes of bulk liquid that must have H_2_ oxidized to the low threshold is difficult, especially with a syntrophic culture, which is continually producing H_2_. In fact, as the syntrophic bacterial population becomes more dependent on the electrode, some studies have indicated that the H_2_ production rate will increase, further driving the electrode oxidation to a point where the maximum oxidation rate will be limited by surface area (Valentine et al., 2000; Adams et al., 2006).

Our modeling efforts indicate that systems below ∼1 mm in distance away from the working electrode will be necessary for culturing syntrophic bacteria. Based on the results, an electrochemical microfluidic reactor appeared to be the ideal system. After developing such a system to test the competition of the methanogenic partner from the co-culture with the electrode, however, the high linear velocities employed within the microfluidic system to maintain laminar flow led to hydraulic retention times (HRTs) of less than 5 min, which washed out our microbes. Even though we were able to maintain anaerobic conditions by placing the microfluidic system in an anaerobic environment (similar to Supplementary Figure S1B), maintaining sterility turned out to be another difficult problem to overcome for us. Therefore, microfluidic systems were not an ideal architecture to enrich for slow-growing syntrophic bacteria.

The electrochemical cell distance from the electrode of 1 mm provided an ideal compromise between a feasible modeling result and engineering design. Thus, we designed a fully autoclavable system that could be operated anaerobically in semi-batch mode with HRTs of a week, and still meet the low H_2_ concentration necessary to achieve a growth advantage for syntrophic bacteria. In this BES system, we controlled the distance away from the electrode between 0.66 and 1.3 mm. When we fed our BES abiotically with H_2_ saturated liquid, current was produced due to the Pt-based activity. When N_2_ was sparged into the H_2_ saturated liquid, which reduced the H_2_ concentration throughout the operating time, the current decreased in unison (results in the Supplementary Material S2, Supplementary Figure S6).

### Bes-Enriching Syntrophic Bacterium From a Separable Co-Culture

We first evaluated the BES through a proof-of-concept study that include competition experiments in simultaneous test and control bioreactors, which were operated in batch mode with volume exchanges performed periodically to add growth medium and to obtain samples. Samples were analyzed for OD_600_, pH, carboxylic acid content, and cell quantification *via* qPCR. For the proof-of-concept we used a co-culture of *S. aciditrophicus* and *M. hungatei*. Both microbes were previously found in co-culture, but have been isolated from one another, and can grow in pure culture (Jackson et al., 1999; McInerney et al., 2007; Mouttaki et al., 2008). *S. aciditrophicus* can grow on crotonic acid in pure culture, but cannot grow in pure culture using any of the other tested carbon sources. In co-culture, however, *S. aciditrophicus* can use a variety of carbon sources, including benzoic acid, which has been used to force *S. aciditrophicus* to grow in co-culture with *M. hungatei*.

Our initial efforts to grow *S. aciditrophicus* in pure culture with crotonic acid in the BES were unsuccessful, likely because the new environment extended the already-long doubling time. We, therefore, tested the co-culture in the BES with benzoic acid as a substrate. We anticipated the syntrophic bacterium to separate from the methanogen, and eventually become enriched even while using benzoic acid as the carbon source. By omitting crotonic acid, which *S. aciditrophicus* could utilize in pure culture, the enriched syntrophic bacterial population would have to be sustained by the oxidation of H_2_ at the electrode. As we performed batch exchanges for the system every 4–5 days, more and more non-growing methanogens should be washed out of the system.

**FIGURE 4:**
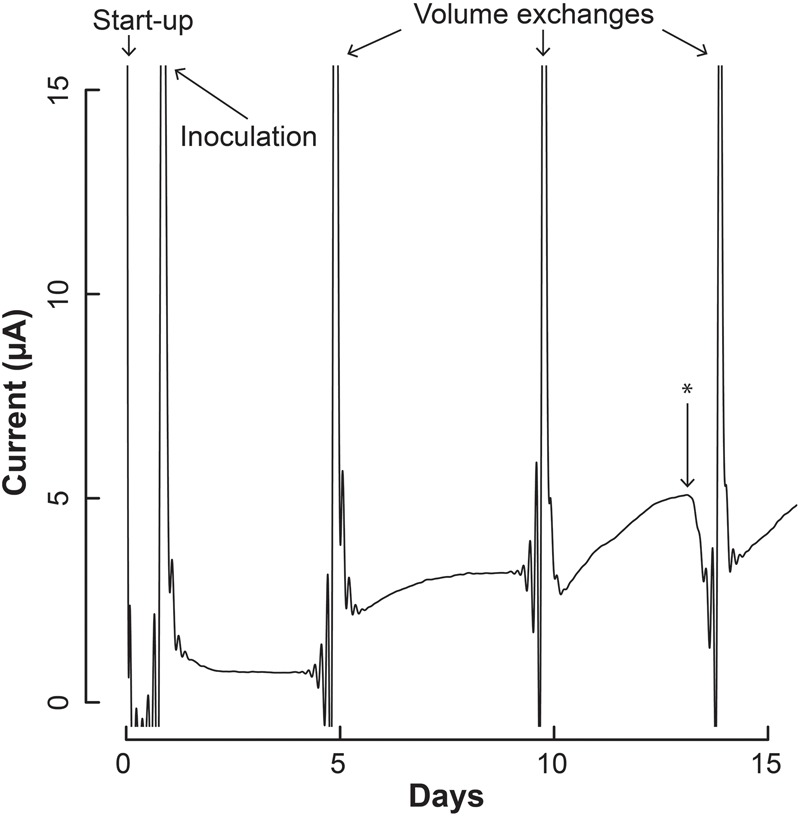
Current production throughout the operating period by the co-culture of *S. aciditrophicus* and *M. hungatei* in the BES. After inoculation, three volume exchanges were performed to add nutrients and obtain samples. Oxygen was introduced to the anaerobic gas on Day 13, which is marked by the asterisk (^∗^).

We inoculated 5 mL of a 1:1 co-culture of *S. aciditrophicus* and *M. hungatei* into the BESs and allowed them to grow for a number of days until the sampling and medium exchange. The BESs were operated in batch mode for which medium exchange would remove more than half of the internal volume that varied between 3 and 5 mL depending on whether the Nafion membrane had stretched into or out of the working electrode chamber. During medium exchanges, 5 mL of fresh sterile *S. aciditrophicus* medium with benzoic acid was added instead of the 1:1 mixture to slow down *M. hungatei* growth further. Upon inoculation, current remained low (Figure 4), which was anticipated because the microbes were still conditioning to each other. After about 5 days, when the first medium exchange had occurred, current began to increase (Figure 4).

The increase in electric current confirmed that the syntrophic bacterium was beginning to provide H_2_ to the electrode, rather than the methanogenic partner. The current increased from a baseline current of about 7.5⋅10^-1^ μA, which occurred at the end of the first batch period (Day 4), to 3.2 μA (Day 5) when new medium was added. After the second medium change, the current reached 5.1 μA (Day 13). Then we added a small amount of oxygen by stopping the flow of anaerobic gas (Day 13) to identify whether the current was truly due to anaerobic microbial activity. Indeed this oxygen exposure quickly decreased the current. Once anaerobic gas was restored during the next medium exchange, the current continued to increase, indicating that the short exposure to oxygen was minimal and did not permanently harm the anaerobic microbial activity. Finally, the slope of the current increases became steeper during the three periods following inoculation from 0.34 μA⋅day^-1^ during the first batch, to 0.92 μA⋅day^-1^ during the second batch, and to 0.99 μA⋅day^-1^ during the final batch. This increasing current rate indicated to us that the microbial H_2_ production rate was increasing during the operating period, implying that the syntrophic bacterial population was also increasing.

**FIGURE 5:**
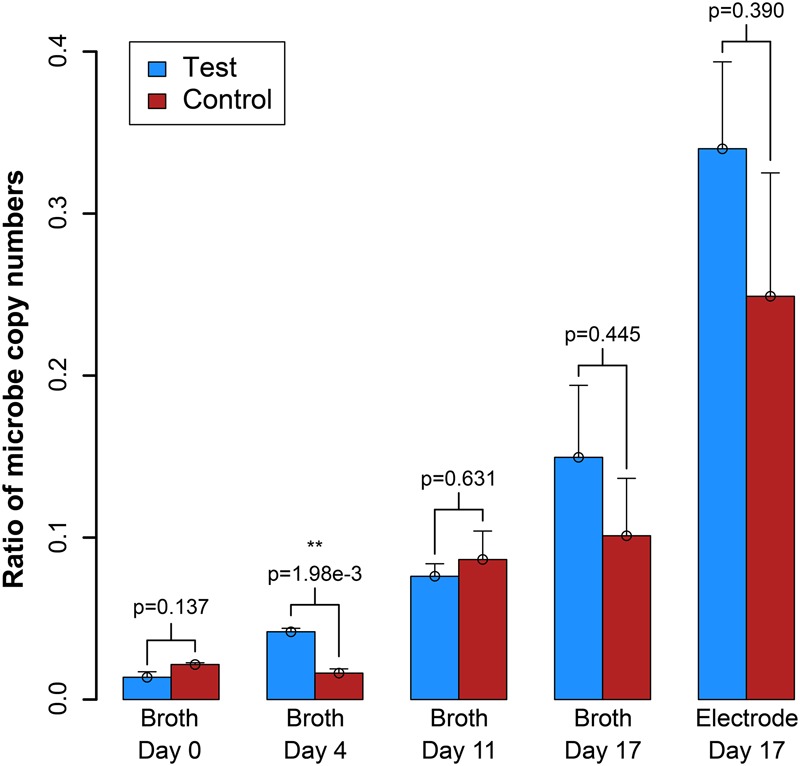
Ratio of the copy number of the syntroph, *S. aciditrophicus*, to the methanogen, *M. hungatei* (syntroph/methanogen). Error bars indicate the standard error from qPCR analysis. ^∗∗^signifies *p*-values <0.01.

We also measured OD_600_ and benzoic acid concentrations for the samples, which did not provide as clear results as the electric current densities (Supplementary Figure S7). However, from a simple evaluation of the conversion of benzoic acid into H_2_, which would be oxidized and measured as electrical current production, we predict a Coulombic efficiency of 37–50%, indicating heavy use of the electrode as a replacement for the methanogen (calculation in Supplementary Material S3). We further measured the DNA for the syntrophic partners with qPCR from the reactor samples, and on the electrode at the end of the experiment (Day 17). After inoculation, the total copy number of cells in the system decreased (Supplementary Figure S8). However, this was anticipated because the doubling time for *S. aciditrophicus* and *M. hungatei* are about a week, which is longer than the periods between medium exchanges. Comparing the ratio of the copy number of the syntrophic bacterium to methanogenic partner in the bioreactors (i.e., syntroph-to-methanogen ratio), we observed that the BES was seemingly enriching for the syntrophic bacterium compared to the methanogenic partner (Figure 5). At inoculation, the test and control BESs had ratios of 1.3⋅10^-2^ and 2.2⋅10^-2^, respectively. In the end of the experiment, the ratio for the test BES increased to 1.4⋅10^-1^, while the ratio increased to 9⋅10^-2^ for the control BES (Figure 5). The electrode showed the most promise for culturing the syntrophic bacterium, because the test electrode possessed a syntroph-to-methanogen ratio of 3.3⋅10^-1^, while this was 2.3⋅10^-1^ for the control electrode. Even though we observed a trend, the qPCR results did not demonstrate a statistically significant difference after Day 4 (Figure 5).

### Bes Test With Inseparable Co-Culture

**FIGURE 6:**
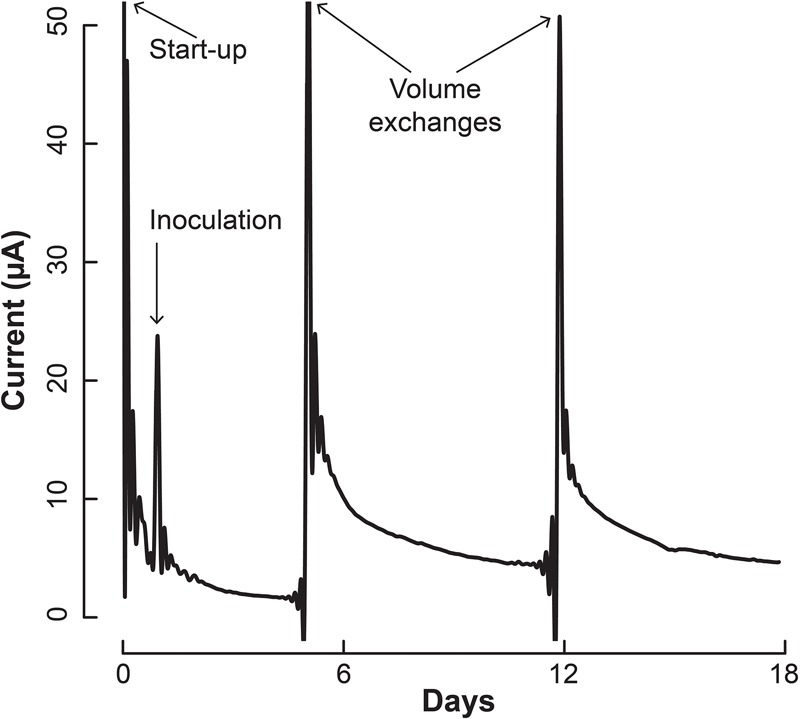
Current profile of the *S. zehnderi* and *M. formicicum* co-culture in the BES.

We also used the system to perform a competition experiment with the never-before-separated co-culture consisting of *S. zehnderi* and *M. formicicum* (Sousa et al., 2007). This co-culture had originally been enriched from a fermentation reactor that was degrading oleic acid. Trials to grow this syntrophic bacterium with crotonic acid have not been successful, and it has only been shown to grow on carboxylic acids (C4 to C18) together with the methanogen. The growth rate of *S. zehnderi* has yet to be determined because it has never been isolated from its *M. formicicum* partner. We inoculated 5 mL of this co-culture into the BES, and performed periodic volume exchanges, using sterile *M. formicicum* medium supplemented with 0.5 mM oleic acid (Figure 6). The current response produced by this co-culture did not show the same response as that with *S. aciditrophicus* and *M. hungatei* (Figure 6). After introduction of new medium, the current quickly increased, likely due to the oxidation of components in the medium, but then reached a baseline of 1.5 μA. At the end of the second batch, the system produced a current of 4.6 μA, while this was 4.8 μA at the end of the third batch. This increase in current through time may provide some indication that there could have been H_2_ transfer from *S. zehnderi* to the electrode in the BES. However, we never observed an electric current density profile that indicated growth. The OD_600_ and pH measurement also did not provide any positive indication about syntrophic bacterial enrichment (Supplementary Figure S9). In addition, oleic acid consumption was not observed, although the slow growth rate of *S. zehnderi* may not yield a measurable difference throughout an operating period of 5 days. In co-culture experiments onset of methane production from oleic acid occurs only after 10–15 days of incubation, when acetate starts also to accumulate. Further efforts to measure degradation by-products, such as carboxylic acids, which was reported by Sousa et al. (2007), were inconclusive, yielding concentrations below the sensitivity level for acetate.

**FIGURE 7:**
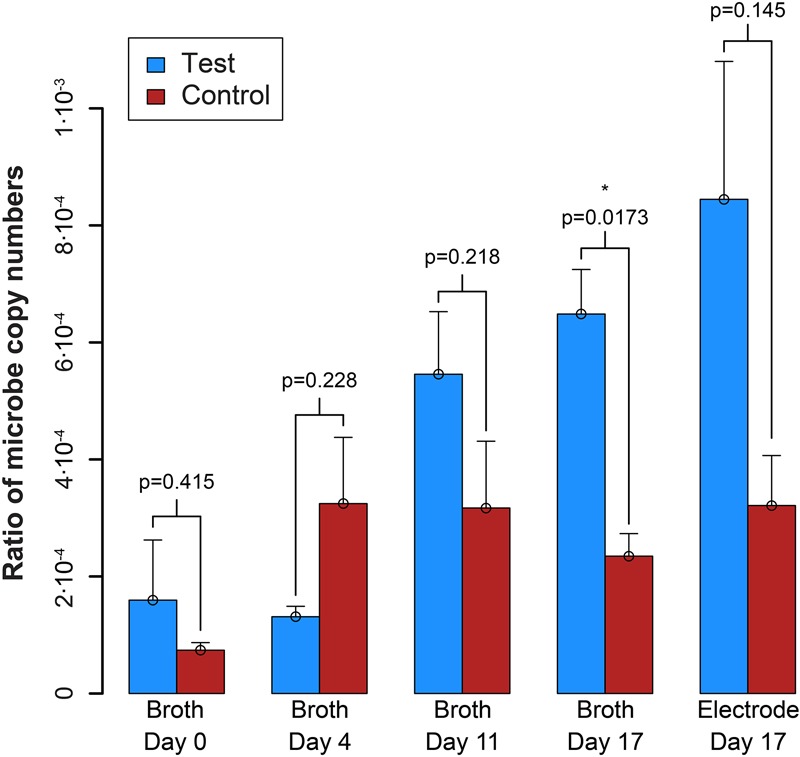
Ratio of the copy number of the syntroph, *S. zehnderi*, to the methanogen, *M. formicicum* (syntroph/methanogen). ^∗^signifies *p*-values <0.05. Error bars indicate the standard error from qPCR analysis.

qPCR results explained the absence of measurable syntrophic bacterial growth because the copy numbers for *S. zehnderi* were low during the experiment and continued to decrease rapidly throughout the operating period. Concurrently, since the H_2_ production from *S. zehnderi* decreased throughout the operating period, *M. formicicum* cell count also decreased, indicating that we were sampling/exchanging the system too often to match the growth rate of the co-culture (Supplementary Figure S10). One benefit of performing such medium exchanges often was that it washed out the competing methanogenic partner, and retains syntrophic bacteria that had settled within the electrode matrix. Indeed, while we observed cell washout during sampling/exchanging, the syntroph-to-methanogen ratio steadily increased throughout the operating period compared to the control (Figure 7). The syntroph-to-methanogen ratio for the control BES remained steady at around 2.5⋅10^-4^. The ratio in the test BES steadily increased from 1.2⋅10^-4^ to 6.4⋅10^-4^, which is a 5.3-fold increase from Day 0 to 17. When the experiment was completed on the 17th day, the difference of the syntroph-to-methanogen ratio between the test and control BES was significant. We observed the same trend for the electrode when it was harvested at Day 17, but due to a larger error in the qPCR data, this difference in the syntroph-to-methanogen ratio was not statistically significant. Since we had started this co-culture experiment with a lawn of the methanogenic partner, the syntroph-to-methanogen ratio was considerably lower than for the previous co-culture experiment (Figure 5 vs. Figure 7).

## Discussion

Monod-growth mathematical modeling indicated that operating below a H_2_ threshold concentration of 51 nM would favor syntrophic bacterial growth compared to that of the methanogenic partner to give the syntrophic bacterium a growth advantage with the overarching goal to obtain enrichments. Next, mathematical modeling of the BES architecture indicated that BESs with less than ∼1-mm distance away from the electrode should be able to maintain such low H_2_ threshold concentrations for enrichment experiments. Indeed, experiments performed here with co-cultures of *S. aciditrophicus* and *M. hungatei* and co-cultures *S. zehnderi* and *M. formicicum* demonstrated promising results that enrichment is possible. However, more work is necessary to achieve statistically relevant data points and to enhance the model with an analysis of well-mixed and continuous flow regimes in addition to the batch analyses reported here. We also found that in the choice between growth and washout, we erred on the washout side by operating with a too short HRT, resulting in low cell numbers. Therefore, an updated BES architecture is necessary with a longer HRT (longer period between sampling/exchanging) as a batch system or with a continuous system when possible, while maintaining the same small maximum distances away from the anode.

We will continue to enrich for syntrophic bacteria from co-cultures with an updated BES, because of the combination of the increasing electric current density for the co-culture of *S. aciditrophicus* and *M. hungatei* throughout the operating period, and the promising trends in the qPCR data for both co-cultures. We further plan to improve our qPCR methods with stronger statistical certainty for the low cell-counts experienced. The ultimate goal is to isolate syntrophic bacteria from natural microbiome samples. Now that we have successfully completed the competition experiments between the methanogenic partner with the electrode as a proof-of-concept study, we can use bromoethylsulfonate to completely inhibit methanogenic growth, while maintaining syntrophic growth to speed up the enrichment. Regardless, we would likely still need to use an agar-plating device that incorporates a working, counter, and reference electrode for total isolation of the syntrophic bacterium into a pure culture (Ueoka et al., 2018).

## Author Contributions

LA proposed the study, provided guidance to JG, and communicated between the research labs. DS provided detailed guidance to the experiments and the growth of syntrophic co-cultures. JG constructed the bioreactor system, designed and ran the model, designed and conducted the experiments, collected samples, and determined analyte and DNA concentrations. LA and JG wrote the manuscript, with revisions from DS.

## Conflict of Interest Statement

The authors declare that the research was conducted in the absence of any commercial or financial relationships that could be construed as a potential conflict of interest.
