# Supplementary material for: Development of a Bioelectrochemical System as a Tool to Enrich H2-Producing Syntrophic Bacteria
Source: Front Microbiol. 2019 Feb 5;10:110. doi: 10.3389/fmicb.2019.00110 (PMC6370660; doi:10.3389/fmicb.2019.00110)
Supplement: Supplementary file 1 [file Data_Sheet_1.pdf]

Supplementary Material

Development of a bioelectrochemical system as a tool to enrich H_2-producing syntrophic bacteria

Juan J.L. Guzman[1], Diana Z. Sousa[2], Largus T. Angenent[3*]

*** Correspondence:** Corresponding Author: l.angenent@uni-tuebingen.de

1 Supplementary Data

1.1 Modeling effort for H_2 concentration threshold to select for syntrophs

We determined the maximum growth rates by analysing serum bottle batch growth of *S. aciditrophicus* and *M. hungatei* in pure culture (**Table S1**). We then calculated the half-saturation constant for H_2-based growth from literature, where we assumed that the final H_2 concentration in the headspace of the serum bottle was the value that inhibited growth (**Table S1**). Finally, we calculated the Monod growth of the syntroph and methanogen in Matlab using the growth terms (**Equation S1 and S2**), and found that the syntrophic bacterial growth curve decreased with increasing H_2, and *vice versa* for the methanogenic partner. Solving for the intersection point of the two growth rate curves yielded a H_2 concentration of 51 nM.

1.2 Abiotic demonstration of H_2 oxidation in BESs

To verify that the system was capable of oxidizing H_2, we performed H_2 dosing tests. We set up a BES continuously recirculating phosphate buffer (PBS) with a serum bottle, and dosed H_2 into the headspace of the serum bottle. Thus, any increase in current observed in the system would be a result of H_2 diffusing from the headspace into the liquid, and being oxidized at the electrode. When we dosed in H_2 into the headspace of the serum bottle, current increased after a small time delay, likely due to allowing H_2 to diffuse into the PBS and for the liquid to be pumped to the electrode (**Figure S6A**). When we sparged the headspace of the bottle with 80%:20% N_2:CO_2 to remove all H_2 in the headspace, the current decreased, as H_2 in the liquid decreased. We then tested the response of the system when H_2 was slowly removed from the system; we refilled the headspace with H_2, then sparged the headspace for short periods of time to gradually decrease the H_2 concentration (**Figure S6B**). With decreasing H_2 concentration in the headspace, we observed lower current produced by the BES. When the sparging was stopped, the current slowed its decrease, but continued to decrease slightly due to continuous H_2 oxidation by the electrode. These abiotic experiments demonstrated that we had designed a reactor, which could oxidize H_2 from liquid solutions.

1.3 Coulombic efficiency calculation of syntrophic benzoate degradation

System volume: 5 mL

Initial concentration of benzoate: 10 mM

Time of batch: 2 days

Ending concentration: 6 mM for test reactor, 7.8 mM for control reactor

Current production: 5.1 uA for test reactor, N/A for control reactor

Reaction stoichiometry: $C_7H_5O_2^- + 7H_2O \rightarrow 3C_2H_3O_2^- + HCO_3^- + 3H_2 + 3H^+$

	Test	Control
Change in benzoate concentration	4 mM	2.2 mM
Change in benzoate total	0.03 mmol	0.01 mmol
H_2 production	0.09 mmol	0.03 mmol
e^- equivalent	0.18 mmol	0.06 mmol
Charge	17.4 C	6.4 V
Current equivalent	10 uA	3.7 uA
Coulombic efficiency	51%	
Current equivalent – Control methanogenesis	6.3 uA	
Effective Coulombic efficiency	37%	

2 Supplementary Equations, Tables, and Figures

2.1 Equations

$$\frac{dpop_{\text{syntroph}}}{dC_{H_2,2}} = \mu_{max,syntroph}\left(1 - \frac{C_{H_2,2}}{k_{half-sat,\text{syntroph}} + C_{H_2,2}}\right)pop_{\text{syntroph}}$$ **Equation S1**

$$\frac{dpop_{\text{methanogen}}}{dC_{H_2,2}} = \mu_{max,methanogen}\left(\frac{C_{H_2,2}}{k_{half-sat,\text{methanogen}} + C_{H_2,2}}\right)pop_{\text{methanogen}}$$ **Equation S2**

$$\frac{dC_{H2}}{dt} = D_{H2}\frac{d^2C_{H_2}}{dz^2} - k_{H_2,oxidation}C_{H_2}$$ **Equation S3**

$$\frac{dC_{H2}}{dt} = D_{H2}\frac{d^2C_1}{dz^2} + \frac{dC_{H2,syntroph}}{dt} + \frac{dC_{H2,methanogen}}{dt}$$ **Equation S4**

$$\frac{dC_{H2,syntroph}}{dt} = C_{syntroph} \cdot k_{H_2,syntroph}$$ **Equation S5**

$$\frac{dC_{H2,methanogen}}{dt} = C_{methanogen} \cdot k_{H_2,methanogen}$$ **Equation S6**

2.2 Tables

Table S1. Parameters used for modelling the growth of *S. aciditrophicus* and *M. hungatei.*

Parameter	Value	Reference
$\mu_{max,syntroph}$	0.002 hr^{-1}	Serum bottle growth data
$k_{half-sat,\mathrm{syntroph}}$	11.90 nM	Calculation using published data (Boone et al., 1989)
$\mu_{max,methanogen}$	0.053 hr^{-1}	Serum bottle growth data
$k_{half-sat,\mathrm{methanogen}}$	665 nM	Calculation using published data (Boone et al., 1989)
D_{H_2}	$4.52 \cdot 10^{-9}\ m^2 \cdot s^{-1}$	(Cussler, 2009)
$k_{H_2,oxidation}$	0.001 $m \cdot s^{-1}$	(Vogel et al., 1975;Jambunathan et al., 2001)
$C_{syntroph}$<br>$C_{methanogen}$	$1 \cdot 10^{-5}$ mM	Initial value, self defined
$C_{H_2,2}$	1000 nM	Initial value, self defined

Table S2. Primers used for qPCR analysis and for building standards for each strain.

Name	Sequence	Reference
S. aciditrophicus	qPCR primers<br>441f: 5'-GGT GGG AAG AAA TGT ATK GA-3'<br>576R: 5'-CTC TTT ACG CCC AAT GAT-3'	(Ziels et al., 2015)
	Standard primers<br>152F: 5'-GGT GGG CTA ATA CCC GAT AAT G-3'<br>824R: 5'-GAT GTT CAC TAG GTG TTG AGG G-3'	Custom designed with NCBI Primer BLAST
M. hungatei	qPCR primers<br>MMB282F: 5'-TCG RTA CGG GTT GTG GG-3'<br>MMB832R: 5'-CAC CTA ACG CRC ATH GTT TAC-3'	(Shin et al., 2010)
	Standard primers<br>176F: 5'- CTG GAA TGT TAT GCG AAC GAA AG-3'<br>969R: 5'-CCT CCT CTC AGC TAG TCA AGT A-3'	Custom designed with NCBI Primer BLAST
S. zhenderi	qPCR primers<br>Synm-678F: 5'-CCW GGT GTA GCG GT-3'<br>Synm-738R: 5'-TCA GGG YCA GTC CAG-3'	(Ziels et al., 2015)
	Standard primers<br>454F: 5'- AGA AGG CCT TAG GGT TGT AAA G-3'<br>1034R: 5'- AGG ATT CGC CAG ATG TCA AG-3'	Custom designed with NCBI Primer BLAST

M. formicicum	qPCR primers<br><br>MBT857F - 5'-CGW AGG GAA GCT GTT AAG T-3'<br>MBT1196R - 5'-TAC CGT CGT CCA CTC CTT-3'	(Ziels et al., 2015)
	Standard primers<br><br>564F: 5’- CCG TGA GAA TTG CTG GAG ATA C-3’<br>1371R: 5’- GAC TAT GGC CTC ATC CAA ACC-3’	Custom designed with NCBI Primer BLAST

2.3 Figures

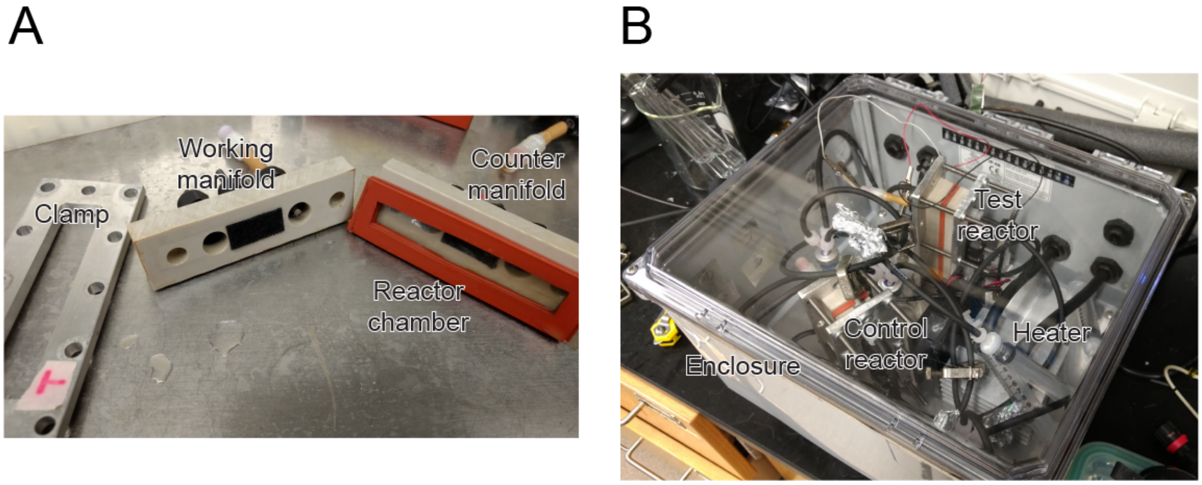

Supplementary Figure 1. Images of the BES experimental system. (A) A teardown shows the tan manifold used to hold the electrodes, red silicon gasket used to form the reactor chamber, clear Nafion membrane sandwiched between the two silicon gaskets, and aluminum clamp used to secure the manifolds together. (B) The enclosure contained both reactors in gas-tight environment with a heater.

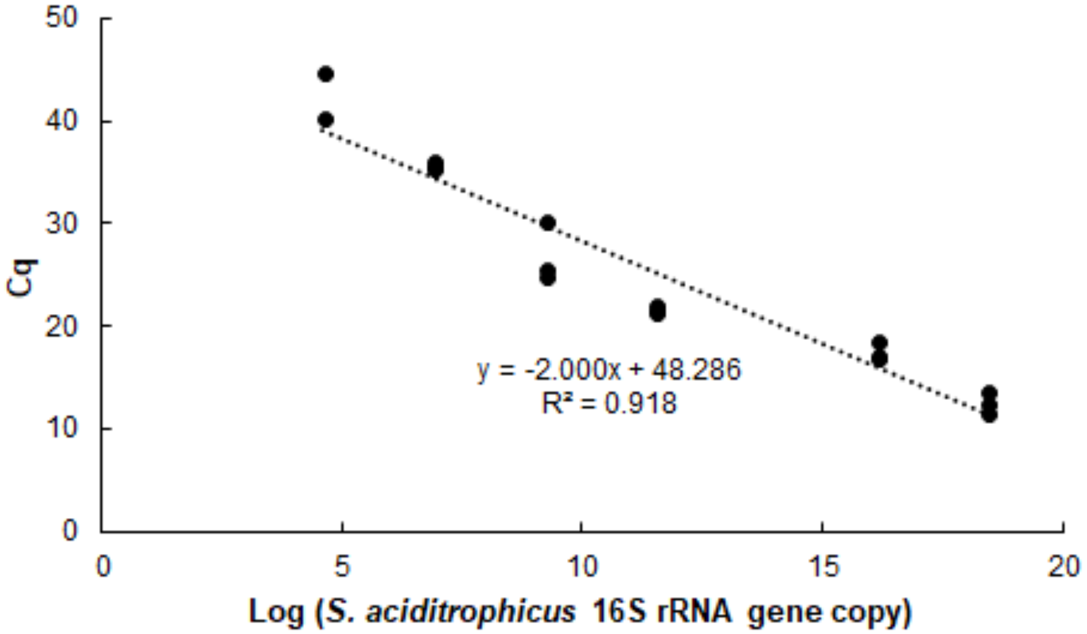

Supplementary Figure 2. qPCR standard curve for *S. aciditrophicus.*

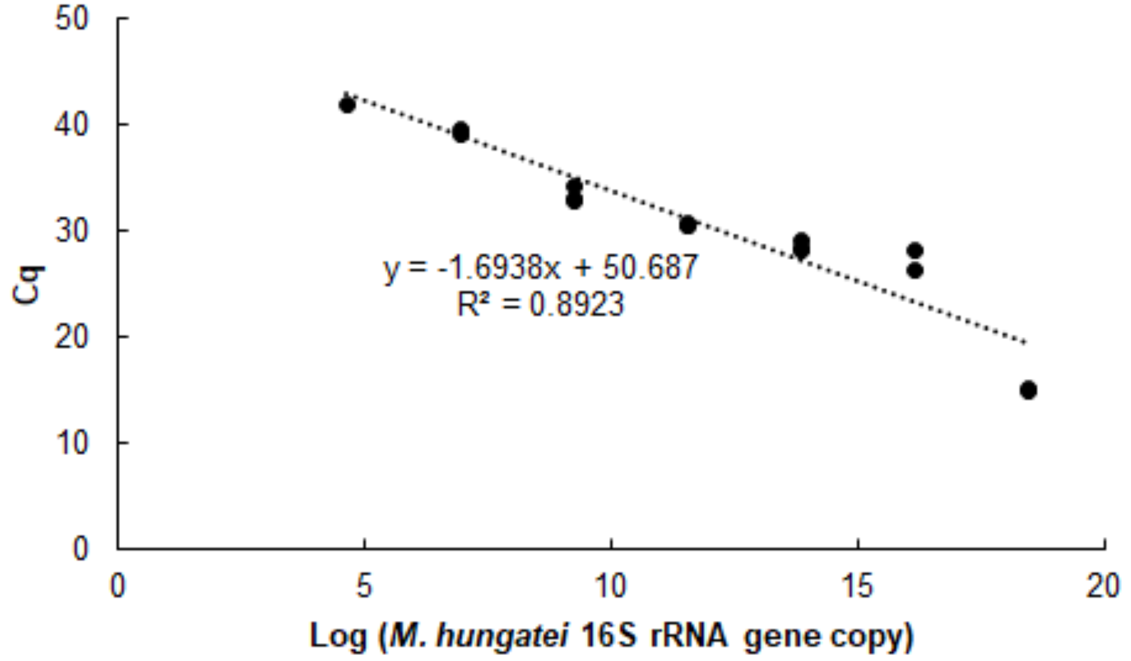

Supplementary Figure 3. qPCR standard curve for *M. hungatei.*

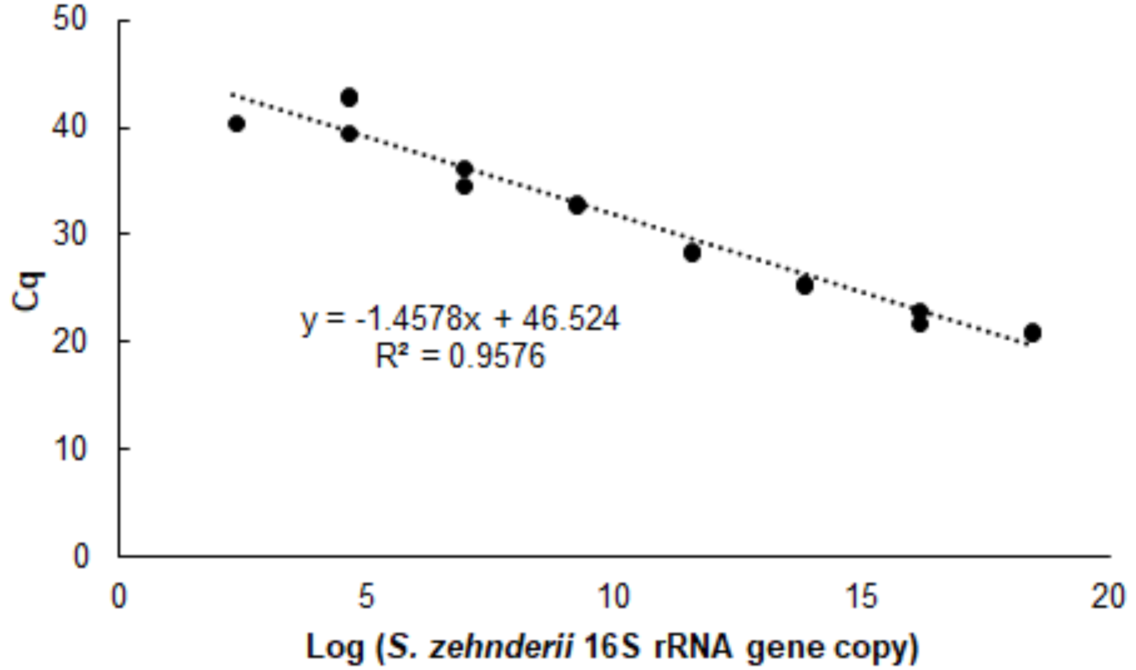

Supplementary Figure 4. qPCR standard curve for *S. zehnderi.*

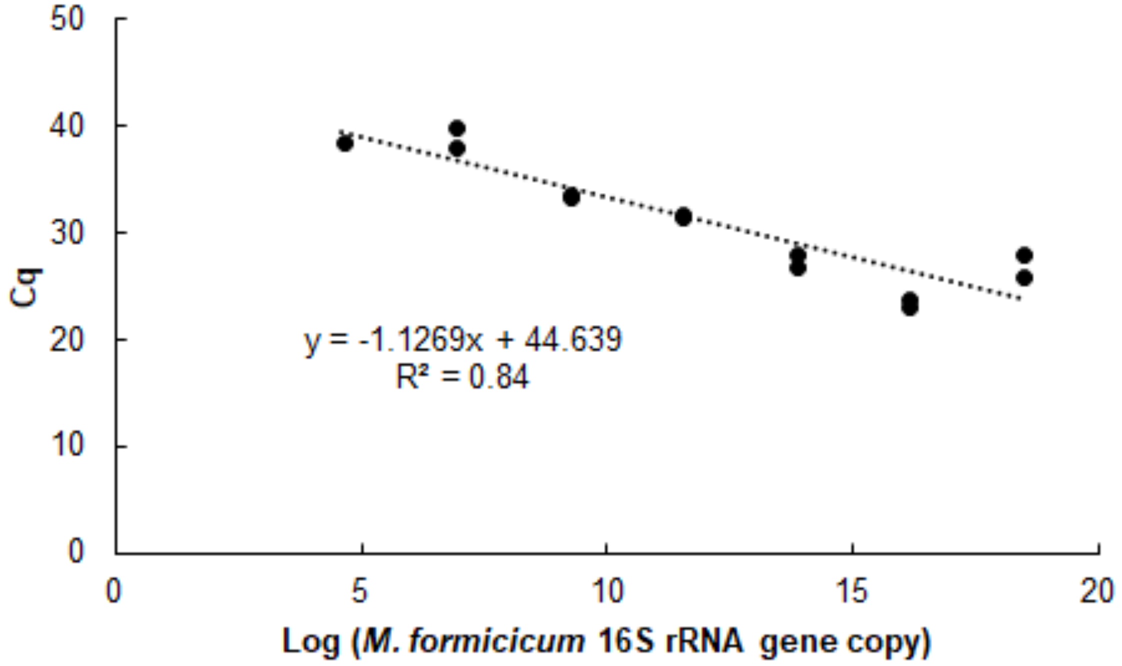

Supplementary Figure 5. qPCR standard curve for *M. formicicum.*

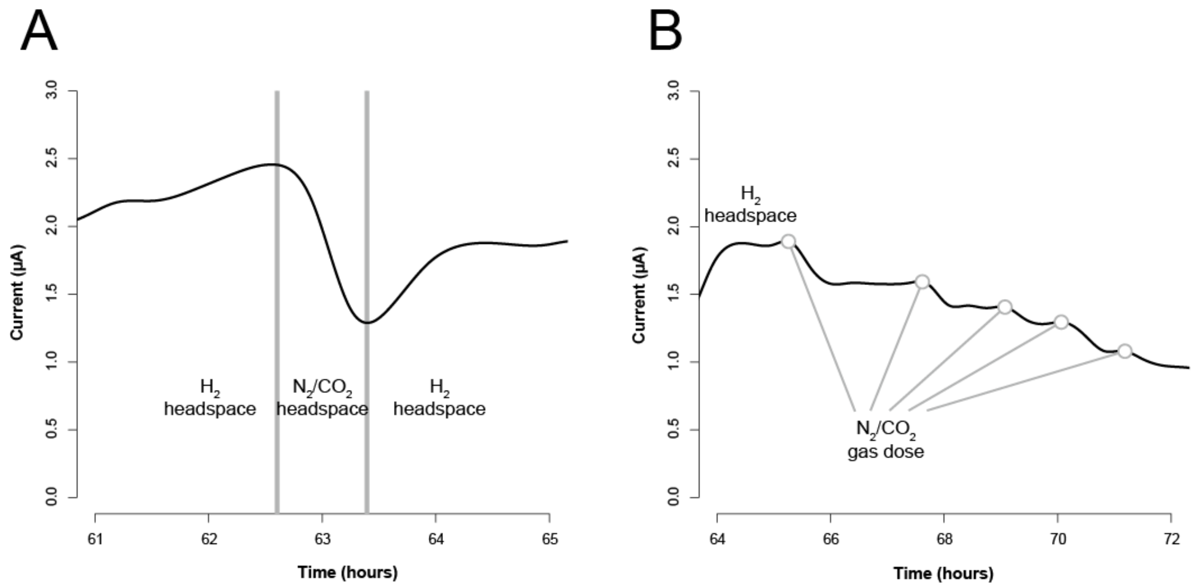

Supplementary Figure 6. Performance of BES system evaluated abiotically through H_2 injections. (A) H_2 was sparged into the headspace of the mixing vessel, replaced with anaerobic gas, and the H_2 was returned. (B) The BES run was initiated with H_2 in the headspace, and anaerobic gas was dosed in, gradually lowering the H_2 concentration.

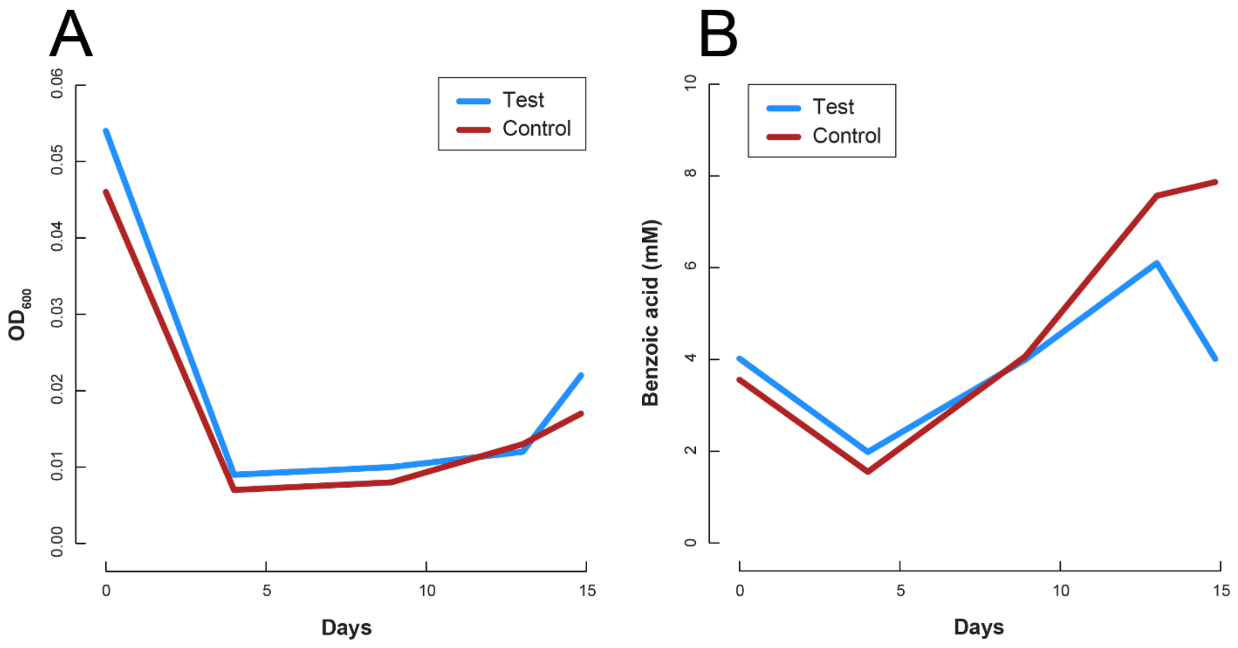

Supplementary Figure 7. (A) OD and (B) benzoic acid profiles during the operating period for the co-culture of *S. aciditrophicus* and *M. hungatei*.

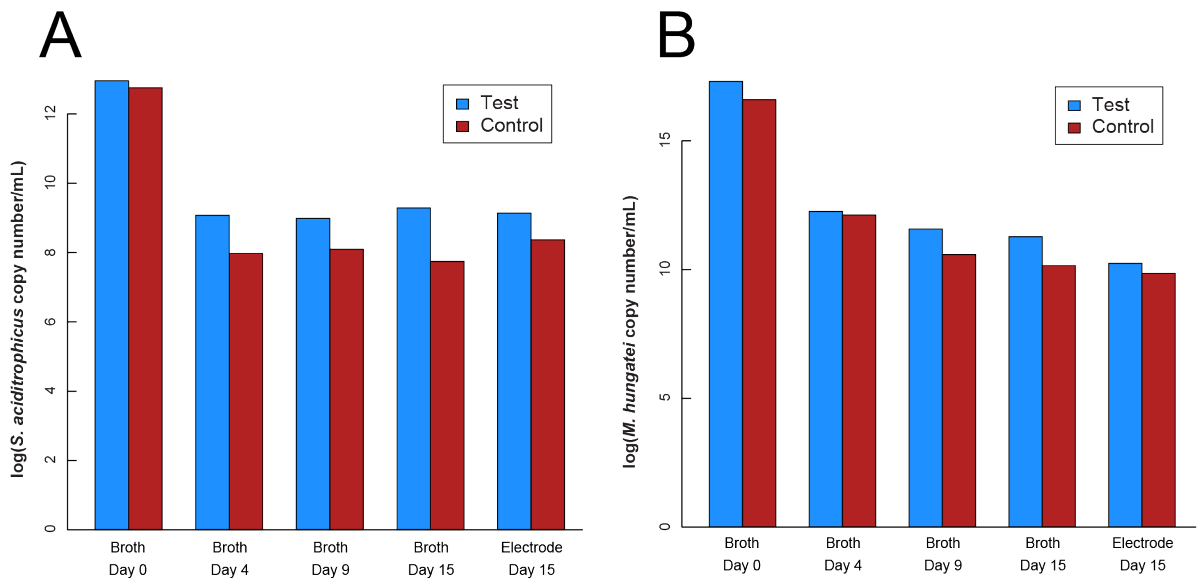

Supplementary Figure 8. Copy number of (A) *S. aciditrophicus* and (B) *M. hungatei* from samples of the broth and the harvested electrode at the end of the experiment. Plotted on a log scale.

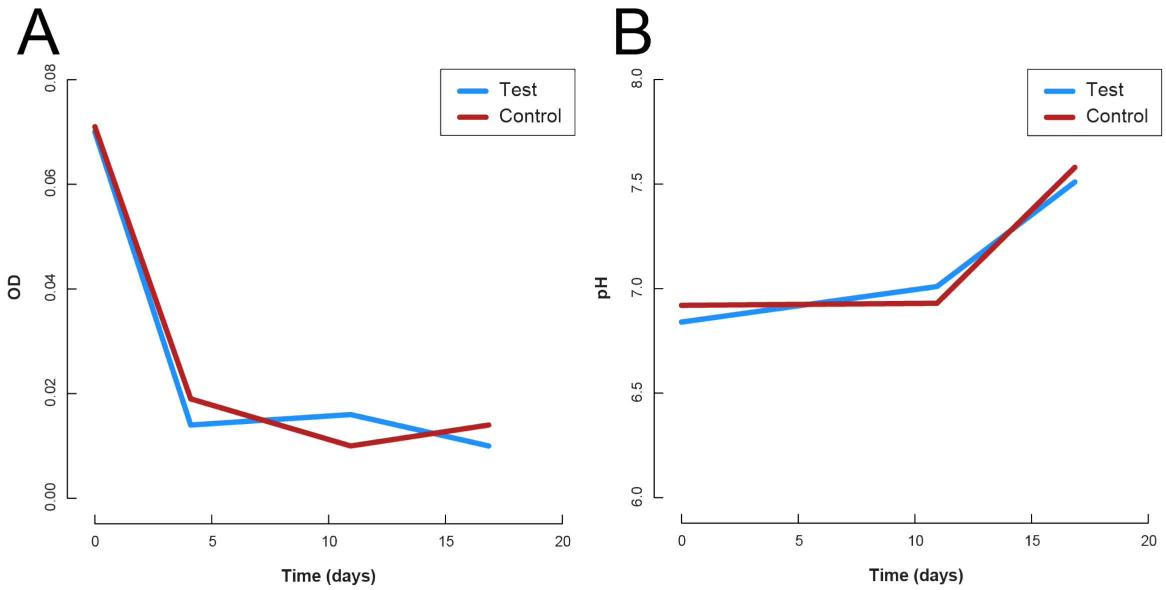

Supplementary Figure 9. OD (A) and pH (B) profiles during the operating period for the co-culture of *S. zehnderi* and *M. formicicum*.

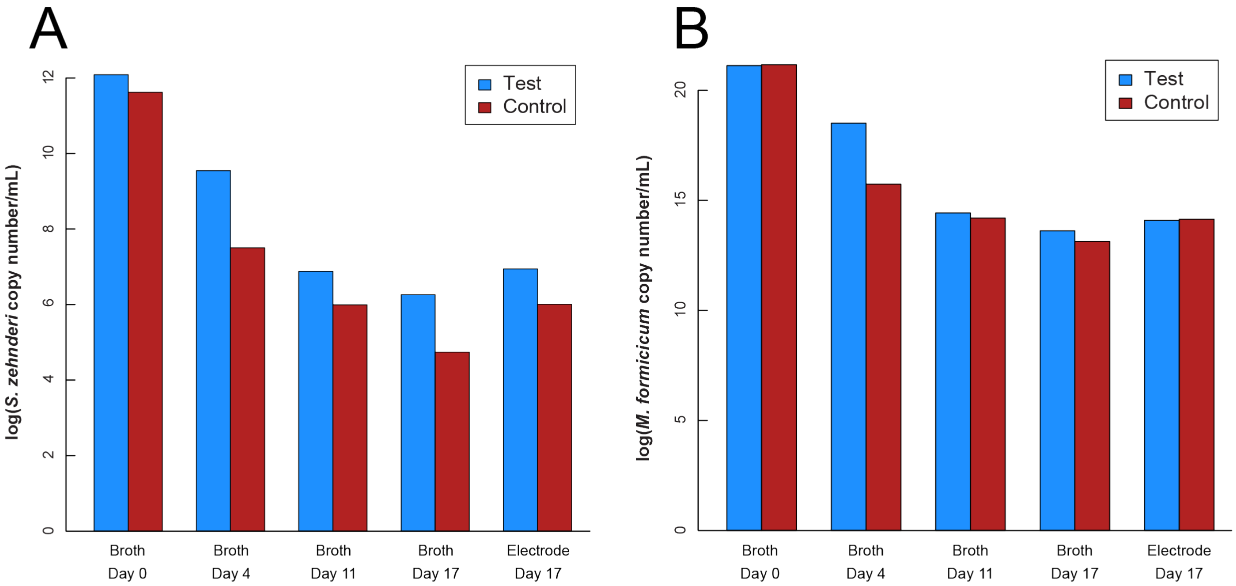

Supplementary Figure 10. Copy number of (A) *S. zehnderi* and (B) *M. formicicum* from samples of the broth and the harvested electrode at the end of the experiment. Plotted on a log scale.

3 References

Boone, D.R., Johnson, R.L., and Liu, Y. (1989). Diffusion of the interspecies electron carriers H_2 and formate in methanogenic ecosystems and its implications in the measurement of K_m for H_2 or formate uptake. *Applied and Environmental Microbiology* 55**,** 1735-1741.

Cussler, E.L. (2009). *Diffusion: mass transfer in fluid systems.* Cambridge university press.

Jambunathan, K., Shah, B.C., Hudson, J.L., and Hillier, A.C. (2001). Scanning electrochemical microscopy of hydrogen electro-oxidation. Rate constant measurements and carbon monoxide poisoning on platinum. *Journal of Electroanalytical Chemistry* 500**,** 279-289. doi: https://doi.org/10.1016/S0022-0728(00)00344-2.

Shin, S.G., Lee, S., Lee, C., Hwang, K., and Hwang, S. (2010). Qualitative and quantitative assessment of microbial community in batch anaerobic digestion of secondary sludge. *Bioresource technology* 101**,** 9461-9470.

Vogel, W., Lundquist, L., Ross, P., and Stonehart, P. (1975). Reaction pathways and poisons—II. *Electrochimica Acta* 20**,** 79-93. doi: http://dx.doi.org/10.1016/0013-4686(75)85048-1.

Ziels, R.M., Beck, D.A., Martí, M., Gough, H.L., Stensel, H.D., and Svensson, B.H. (2015). Monitoring the dynamics of syntrophic β-oxidizing bacteria during anaerobic degradation of oleic acid by quantitative PCR. *FEMS microbiology ecology* 91**,** fiv028.
